# Editorial: Dopaminergic Alterations in Schizophrenia

**DOI:** 10.3389/fnins.2021.663245

**Published:** 2021-03-11

**Authors:** Mark D. Tricklebank, Carol Tamminga, Andrew Grottick, Pierre M. Llorca, Silvia Gatti McArthur, Jean-Claude Martel

**Affiliations:** ^1^King's College London, London, United Kingdom; ^2^UT South Western Medical Center, Department of Psychiatry, Dallas, TX, United States; ^3^Beacon Discovery, San Diego, CA, United States; ^4^Department of Psychiatry, CHU, Clermont-Ferrand, France; ^5^McArthur and Associates GmbH, Basel, Switzerland; ^6^Université du Québec en Abitibi Témiscamingue, Rouyn-Noranda, QC, Canada

**Keywords:** schizophrenia, dopamine, antipsychotics, PDE10 inhibition, neurocircuits

In 1952 Deniker and Delay at St. Anne hospital in Paris conducted a small clinical trial with chlorpromazine. This trial confirmed its outstanding value as a tranquiliser for agitated psychotic patients and it opened the door to understanding the biological basis of schizophrenia (Madras, 2013). Within 10 years a number of structurally distinct and efficacious antipsychotics had been discovered. In 1963, Carlsson and Lindqvist demonstrated that chlorpromazine increased the metabolism of dopamine (Carlsson and Lindqvist, 1963). The true breakthrough on the dopamine hypothesis of schizophrenia came when Snyder's lab demonstrated an association between the treatment of psychosis and the pharmacological manipulation of catecholamine receptors (Snyder et al., 1974). More importantly they went on to show that dopamine receptor binding predicts the clinical and pharmacological potencies of different antipsychotics in humans (Creese et al., 1976a,b). Seeman et al. independently demonstrated the clinical potency of these compounds to correlate with their ability to displace [^3^H]Haloperidol from brain membranes leading to the publication of perhaps the most famous graph in schizophrenia therapeutics (Seeman et al., 1976).

While antipsychotics effectively improve the management of the psychotic symptoms of schizophrenia, cognitive deficits, and negative symptoms have remained major treatment challenges. Side effects of antipsychotics in many individuals are also a source of concern since they could lead to medication interruption (Correll and Kane, 2020). This has forced a re-imagining of the dopamine hypothesis of the disease while looking for new therapies.

In the first chapter of this issue, Conn et al. focus on the role dopamine plays in the cognitive deficits and altered decision making processes in schizophrenia. They also describe the complexities of relevant animal model design. The media attention received by this review may be an indirect indication of the critical relevance of these aspects of the disease for the daily life of patients.

We then come right up to date with a consideration of the utility of patient derived pluripotent stem cells to provide “the disease in a dish” a still, much under-utilized approach (in our view). What is also important in the work of Collo et al. is the systematic attention to the negative symptoms of schizophrenia and the relevant motivational aspects of dopamine neurocircuitries.

This is complemented by the work of Ashton and Jagannath, who review the important role that dopaminergic systems play in circadian rhythm disturbances in schizophrenia, which are considered a pathophysiological hallmark of the disease.

The proposed model of neurocircuit alterations in schizophrenia is centered on hyperactive hippocampal and prefrontal inputs and the role of the ventral tegmental area, as described by the comprehensive work of Sonnenschein et al. that convincingly includes the impact of stressors on these circuits.

The work of Vidal and Pacheco address the most recent advancements in our understanding of dysregulated immune system in schizophrenia. This approach will surely receive much greater attention as more neuro-immunological tools become available. The immune response at critical developmental phases has long been recognized as a major risk factor for schizophrenia, and this concept has been also reviewed in two Frontiers Research Topics (Köhler-Forsberg et al.; Sánchez-Ramón et al.). The dopaminergic system is clearly involved with sensitization processes, which fits with a contributory role in mechanisms of neuronal network reshaping.

The main focus of the present issue is on therapeutic approaches. Understanding the multidimensional phenomenon of medication adherence attitudes in schizophrenia is therefore of paramount importance. Current dopaminergic antipsychotics are still the best therapeutic option, but rates of non-adherence, ranging from 1 to 81%, demonstrate the continuing need for improved therapeutics. El Abdellati et al. review how treatment adherence should be better monitored. They also highlight areas for improvement which include by encouraging people with psychosis to stop cannabis use and providing active support to them by family and medical professionals.

An interesting longitudinal imaging study by Andersen et al. suggests that increases in striatal volume in antipsychotic-naïve first episode people with schizophrenia correlates with how well positive symptoms are controlled with amisulpride. This study represents a link to another interesting issue of Frontiers of Psychiatry (Vita et al.) dedicated to trajectories of brain abnormalities in early schizophrenia.

Are there better ways to manipulate the dopaminergic system to improve functional outcomes in schizophrenia? The final three contributions from Kozak et al., Menniti et al., and Martel and Gatti McArthur, all focus on therapeutic approaches: D1 agonists, new monoaminergic ligands, and PDE10 inhibitors which remain among the best strategies for novel therapeutics.

One answer may come from more selective dopaminergic drugs (including trace amines) acting on dopaminergic subsystems as reviewed in Martel and Gatti-McArthur or, as suggested by the interesting work of Kozak et al. from new D1 selective agonists that show promising pro-cognitive potentials.

Perhaps the greatest challenge comes from the antipsychotic potential of phosphodiesterase PDE10 inhibitors. Why do these compounds demonstrate preclinical properties indicative of efficacious antipsychotic drugs, i.e., are functional D2 antagonists but have no affinity for D2 receptors and do not have any demonstrable clinical benefit? No simple conclusion is reached but the contribution of Menniti et al. highlights the importance of maintaining scientific analysis of these interesting new compounds. The work also questions the translational relevance of conditioned avoidance responding, a test in which all antipsychotics including PDE10 inhibitors are active. It is hoped that withdrawal of the pharmaceutical companies responsible for these compounds from psychiatric drug discovery will not impede such important investigations of translational validity.

We are indebted to all contributing authors, that have carefully crafted this update on the dopaminergic hypothesis in schizophrenia. This hypothesis, with all related questions seems still relevant 50 years on. But much still remains to be discovered. An admirable and worthy calling for those wishing to emulate Delay, Deniker, Carlsson, Seeman, and Snyder.

## Author Contributions

All authors listed have made a substantial, direct and intellectual contribution to the work, and approved it for publication.

## Conflict of Interest

SG is a co-owner of McArthur and Associates GmbH Basel Switzerland; and AG is an employee of Beacon Discovery, San Diego, CA, USA. The remaining authors declare that the research was conducted in the absence of any commercial or financial relationships that could be construed as a potential conflict of interest.
